# Best timing of bilateral knee arthroplasty– an analysis of revision and mortality rates from the German Arthroplasty Registry (EPRD)

**DOI:** 10.1186/s12891-025-08548-5

**Published:** 2025-03-31

**Authors:** Anne Elisabeth Postler, Paula Krull, Yinan Wu, Klaus-Peter Günther, Oliver Melsheimer, Arnd Steinbrück, Jörg Lützner

**Affiliations:** 1 University Center of Orthopaedics, Trauma and Plastic Surgery, University Hospital Carl Gustav Carus, TUD Dresden University of Technology, Fetscherstr. 74, 01307, Dresden, Germany; 2 German Arthroplasty Registry (EPRD), Berlin, Germany

**Keywords:** Total knee arthroplasty, Unicondylar knee arthroplasty, Bilateral, Same day, Simultaneous, Mortality, Revision

## Abstract

**Background:**

The burden of osteoarthritis (OA) in multiple joints is high. For patients with bilateral knee OA there is no clear recommendation when to time the second surgery. The purpose of this study was therefore to compare revision and mortality rate in bilateral unicondylar and bicondylar knee arthroplasties after different strategies of surgical timing in bilateral knee OA from the German Arthroplasty Registry (EPRD).

**Methods:**

Data from the German Arthroplasty Registry (EPRD) was used. Since 2012 a total of 15,154 patients had bilateral knee arthroplasty within one year. Patellofemoral arthroplasties and constraint total knee arthroplasties (TKA) were excluded. 1,144 TKA and 682 unicondylar arthroplasties (UKA) were simultaneously performed, 772 TKA and 292 UKA between 1 and 90 days (short interval) and 24,496 TKA and 2,922 UKA between 91 and 365 days (intermediate interval). Revision and mortality rates were analyzed up to 7 years after surgery. Cox regression was performed to evaluate the influence of different patient characteristics on these outcomes.

**Results:**

The highest cumulative revision rate for any of the bilateral TKA was found for simultaneous surgery with 3.4% (95% CI 2.1–5.5). Lower risk for revision was seen in two-staged surgery in short interval (HR 0.42; 95% CI 0.20–0.90) and intermediate interval (HR 0.58; 95% CI 0.39–0.85). The cumulative one year mortality rate for TKA was comparable in all three groups with 0.8% for simultaneous TKA, 1.3% for short interval two-staged and 0.7% for intermediate interval. In UKA there were no differences between the groups regarding cumulative revision rate and mortality rate.

**Conclusion:**

TKA should be performed simultaneously in selected patients only, the two-staged procedure demonstrated lower revision risks. For UKA we found no differences in timing, simultaneous surgery seems to be a safe option.

**Trial registration:**

Clinical trial number not applicable.

**Level of evidence:**

III.

**Supplementary Information:**

The online version contains supplementary material available at 10.1186/s12891-025-08548-5.

## Background

Osteoarthritis (OA) of the knee is a frequent cause of functional impairment. The FDA has recently categorized knee OA as a serious disease [15, 16]. Total knee arthroplasty (TKA) is a very effective treatment option for advanced OA of the knee, which decreases pain and improves function [18]. Evans et al. reported about an expected survival time (time to revision or death) of TKA up to 20 years in around 90.1% of patients [6]. Bilateral OA of the knee ranges from 60 to 90.4% [4, 5, 12, 25]. The burden of OA in multiple joints is high [5] and multiple surgeries and anesthesia procedures need a prolonged rehabilitation and recovery. In cases of bilateral knee OA there is always the question whether TKA is necessary bilateral and if so, how it should be performed (one stage, two stages, time between stages). Most patients get bilateral TKA as a staged procedure after a convalescence of several months. The one-staged, same day bilateral TKA is rather reserved for younger patients with less comorbidities [3] and risks and benefits are discussed controversially. Multiple single-center and retrospective studies concluded bilateral TKA as safe procedures in specialized high-volume center [22], but reserved for selected patients [1] and considering higher complications rates [2, 3, 9, 11, 19, 20, 24, 27]. In an older systematic review of retrospective studies Fu et al. reported about significant higher 30-days-mortality as well as pulmonary embolism and blood transfusion rate following same day bilateral TKA, but lower rates of deep infection and revision [9]. An recent meta-analysis showed significantly over twofold increased 90-day-mortality for same day bilateral TKA compared to staged bilateral TKA with equivocal revision rate within the first year and different trends of separate complication rates [20]. Another systematic review with contemporary studies demonstrated no differences if the baseline characteristics of the patients were similar [8, 21].

Aim of this study was therefore (1) to investigate the frequency and timing of bilateral TKA in Germany and (2) to determine revision and mortality rates associated with simultaneous versus two-staged TKA in the German Arthroplasty Registry (EPRD).

## Methods

In this retrospective registry study data acquisition started in November 2012 and includes currently a total number of more than 3 million hip and knee replacements in its database. The EPRD covers primary and revision arthroplasty surgeries. The EPRD covers 70% of all hip and knee arthroplasties in Germany [10]. Once entered into the registry and insured with a participating insurance company, the follow-up of an arthroplasty is nearly complete because data on revisions is obtained not only by hospitals, but additionally by health insurance companies. Demographic data such as age, sex, body mass index as well as comorbidities (weighted Elixhauser Score), length of stay and readmission rates are documented. Death and revision data are obtained from health insurance companies on a regular basis [14]. Due to the voluntary nature of the EPRD, a significant proportion of hospitals do not participate in the registry, leading to a systematic underrepresentation of certain healthcare providers, accounting for approximately 30% of missing data. Furthermore, patients who are not insured with a participating insurance company are not included in the follow-up analyses. Revisions and mortality were analyzed of all patients from 12/2012 until 31/2023. Annual hospital volume is documented as well. High-volume center for TKA/UKA surgeries were defined as performing at least 500 TKA/150 UKA per year.

From a total number of 377,897 patients with knee arthroplasty in cases of OA (ICD-10 code diagnoses of M17.-) which were registered in the EPRD from 2012, 40,535 could be identified as having a bilateral primary knee arthroplasty and 15,154 within one year. Patients with a second knee arthroplasty due to other diagnosis than OA (M17), unknown implant types, patellofemoral arthroplasty and constraint TKA were not included. TKA and UKA were separately analyzed.

**Fig. 1:**
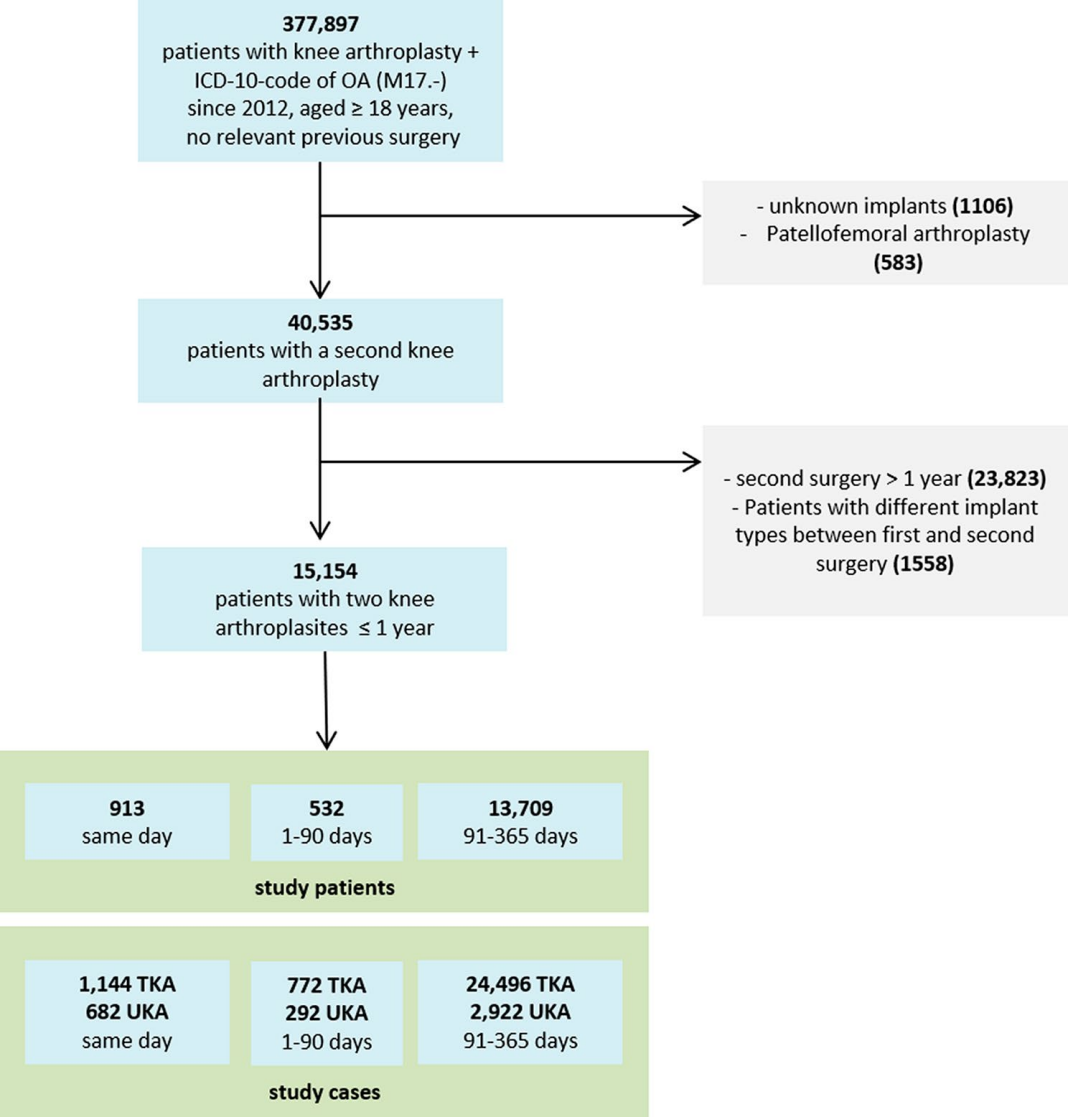
Flowchart

Three different time intervals between both knee arthroplasties were chosen: (1) both procedures on exactly the same day (simultaneous) with 1,144 TKA and 682 UKA, (2) staged surgery with an interval of one to 90 days between index surgery and second knee arthroplasty (short interval) with 772 TKA and 292 UKA and (3) staged surgery with an interval of 91 to 365 days between both procedures (intermediate interval) with 24,496 TKA and 2,922 UKA, see flowchart, Fig. 1.

Between the groups there were significant differences regarding age, sex, BMI in categories and comorbidities (weighted Elixhauser Index). The endpoints were revision surgeries and death of the patients.

The characteristics of the study cases are summarized in Table 1 and supplementary Figs. 6 and 7.

**Table 1:** Characteristics of study cases

	TKA				UKA			
	same day	1–90 d	91–365 d	*p*-value	same day	1–90 d	91–365 d	*p*-value
	(*n* = 1,144)	(*n* = 772)	(*n* = 24,496)		(*n* = 682)	(*n* = 292)	(*n* = 2,922)	
**Sex**								
Female	630 (55%)	372 (48%)	15,361 (63%)	**< 0.001**	324 (48%)	120 (41%)	1,486 (51%)	**0.003**
Male	514 (45%)	400 (52%)	9,135 (37%)		358 (52%)	172 (59%)	1,436 (49%)	
**Age**								
< 65	386 (34%)	345 (45%)	8,638 (35%)	0.063	352 (52%)	184 (63%)	1,675 (57%)	**< 0.001**
65–74	476 (42%)	232 (30%)	8,547 (35%)		238 (35%)	84 (29%)	819 (28%)	
75–84	262 (23%)	177 (23%)	6,774 (28%)		90 (13%)	24 (8.2%)	414 (14%)	
85+	20 (1.7%)	18 (2.3%)	537 (2.2%)		2 (0.3%)	0	14 (0.5%)	
**BMI**								
mean (SD)	30.0 (5.6)	31.6 (6.2)	31.9 (6.3)	**< 0.001**	30.5 (5.1)	31.3 (5.2)	30.9 (5.5)	0.15
**weighted Elixhauser Score by VW**								
mean (SD)	0.6 (3.7)	0.4 (4.0)	0.5 (4.0)	**< 0.001**	-0.24 (2.93)	-0.30 (3.24)	-0.04 (3.21)	0.2
**Annual hospital volume [TKA/UKA]**								
< 250/50	294 (26%)	314 (41%)	13,255 (55%)	**< 0.001**	78 (12%)	57 (20%)	1,163 (41%)	**< 0.001**
251/51–500/150	622 (55%)	124 (16%)	5,403 (23%)		144 (21%)	26 (9.3%)	610 (22%)	
> 500/150	224 (20%)	322 (42%)	5,302 (22%)		454 (67%)	196 (70%)	1,037 (37%)	
missing	4	12	536		6	13	112	

*One-way ANOVA for continuous variables (e.g. age at admission), Chi-squared test for categorical variables (e.g. sex of patient)

### Statistical analysis

Data description was based on means and standard deviation (SD) for continuous variables and absolute and relative frequencies for categorical variables. To evaluate the influence of different patient characteristics on revision and mortality, a Cox regression model was applied.

Cumulative incidences for the endpoints death of the patient and revision of each arthroplasty separately were calculated with the Kaplan-Meier survival function. A pairwise Log-Rank test with Holm´s correction for multiple testing was applied to identify intergroup differences.

A p-value threshold of 0.05 was considered statistically significant. All data analyses were carried out using R statistical software, Version R-4.2.0 (R Foundation for Statistical Computing, Vienna, Austria).

## Results

**Fig. 2:**
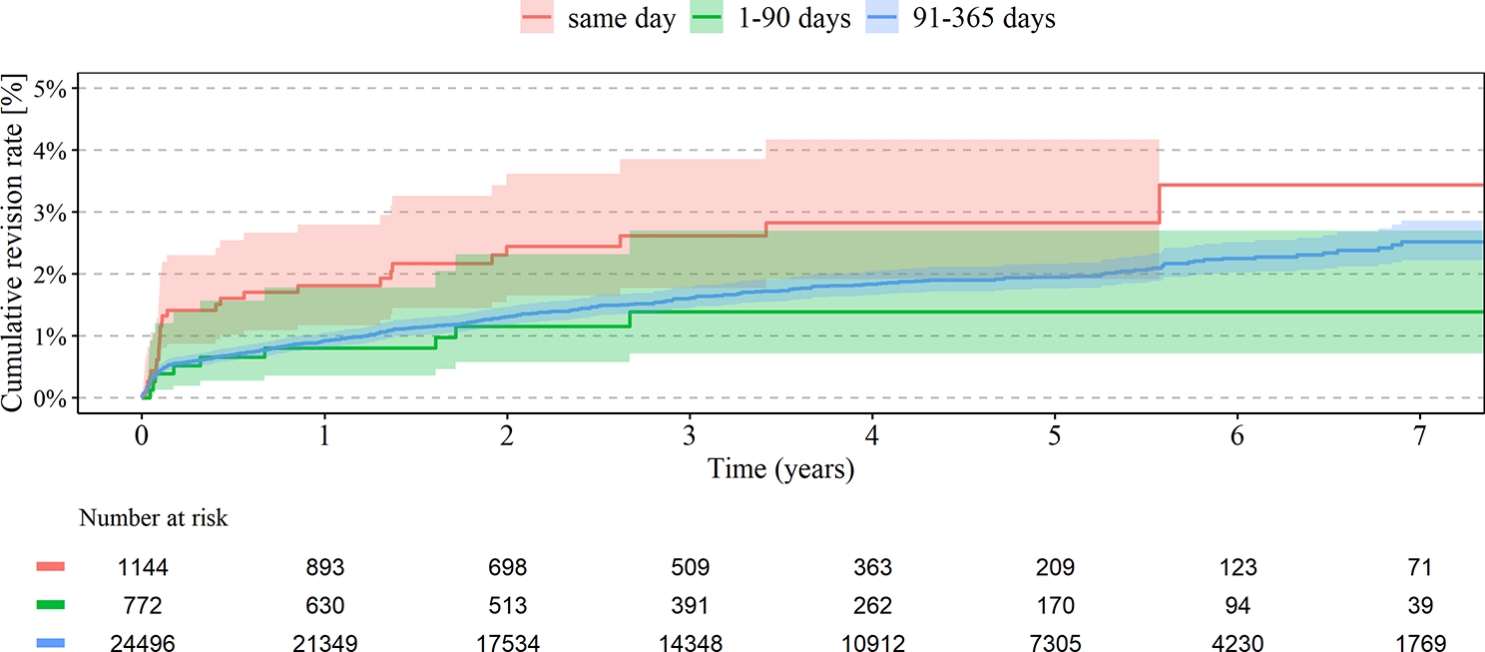
Revision rate in TKA

**Fig. 3:**
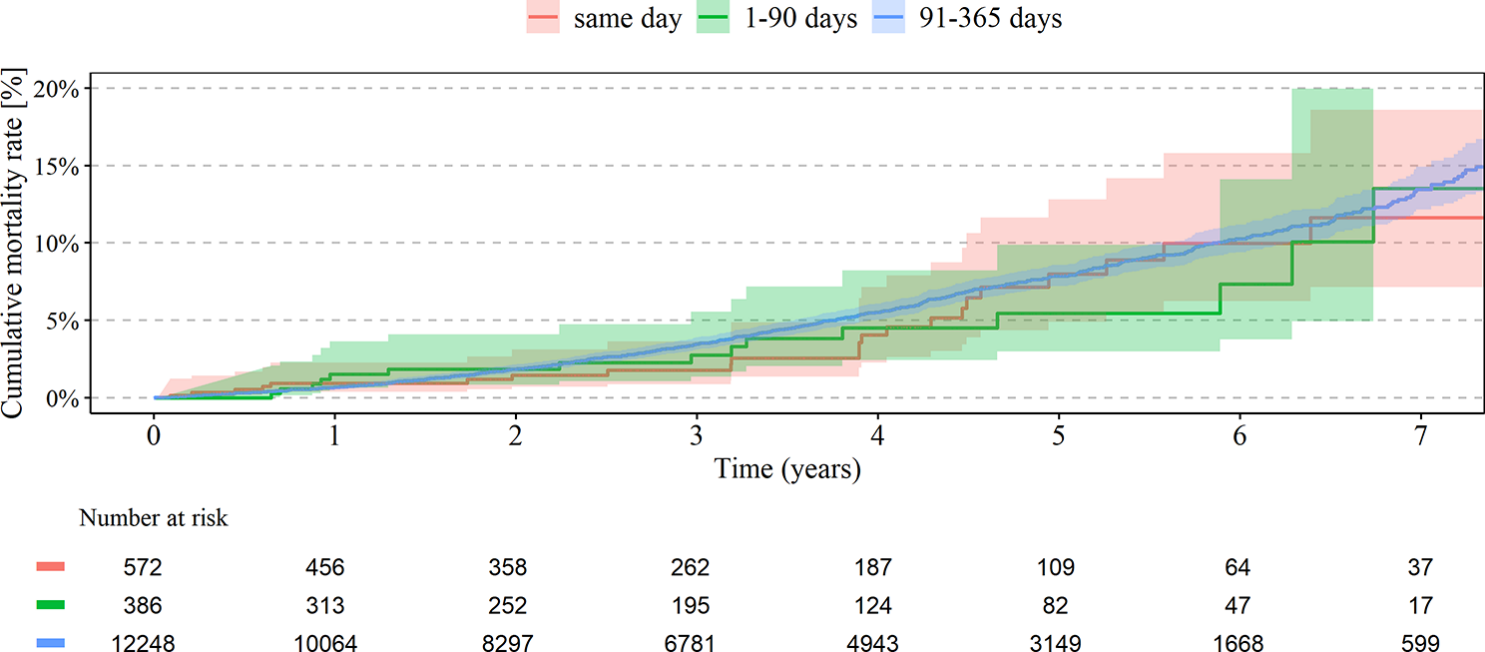
Mortality rate in TKA

A total of 10.7% of patients with primary knee arthroplastyhave had both knee joints replaced, 4.0% in the same year and 0.2% simultaneously. The analysis demonstrated significantly higher cumulative revision rates for any of the bilateral TKA within 7 years for simultaneous (3.4%) vs. short (1.4%, HR 0.42; 95% CI 0.20–0.90) and intermediate interval (2.5%, HR 0.58; 95% CI 0.39–0.85), see Table 2; Fig. 2. Lower risk for revision was recognized if the surgery was performed in a high-volume center with more than 500 knee arthroplasties per year (HR 0.77; 95% CI 0.64–1.02) and for patients between 65 and 74 years (HR 0.61; 95% CI 0.49–0.77) Higher risk for revision was found for higher weighted Elixhauser Index (HR 1.03 per point; 95% CI 1.01–1.06). No significant association was found with sex and BMI, see Table 4. For TKA, there was no significant difference between the cumulative mortality rate in every group, see Table 3; Fig. 3. Higher risk for mortality in bilateral TKA was seen in men (HR 1.81; 95% CI 1.56–2.11), BMI > 40 kg/m^2^ (HR 2.1 compared to normal weight; 95% CI 1.34–3.29) and higher Elixhauser Index (HR 1.06 per point; 95%CI 1.04–1.87). Lower risk for mortality in bilateral TKA was seen in high-volume centers with > 500/y (HR 0.73; 95% CI 0.59–0.90), see Table 4.

**Table 2:** Cumulative events for revision (95% confidence Interval) in *TKA* and *UKA*

**TKA**	**1 year**	**3 years**	**5 years**	**7 years**
same day (%)	1.8 (1.2–2.8)	2.6 (1.8–3.8)	2.8 (1.9–4.2)	3.4 (2.1–5.5)
1–90 days (%)	0.8 (0.4–1.8)	1.4 (0.7–2.7)	1.4 (0.7–2.7)	1.4 (0.7–2.7)
91–365 days (%)	0.9 (0.8–1.1)	1.6 (1.4–1.8)	2 (1.8–2.2)	2.5 (2.2–2.9)
**UKA**	**1 year**	**3 years**	**5 years**	**7 years**
same day (%)	1.8 (1.0-3.2)	3.4 (2.2–6.2)	4.2 (2.7–6.4)	5.8 (3.1–10.7)
1–90 days (%)	1.1 (0.4–3.5)	2.8 (1.2–6.2)	4.2 (2.1–8.5)	4.2 (2.1–8.5)
91–365 days (%)	1.5 (1.1.-2.0)	2.3 (1.8-3.0)	3.0 (2.3–3.8)	3.1 (2.4–4.1)

**Fig. 4:**
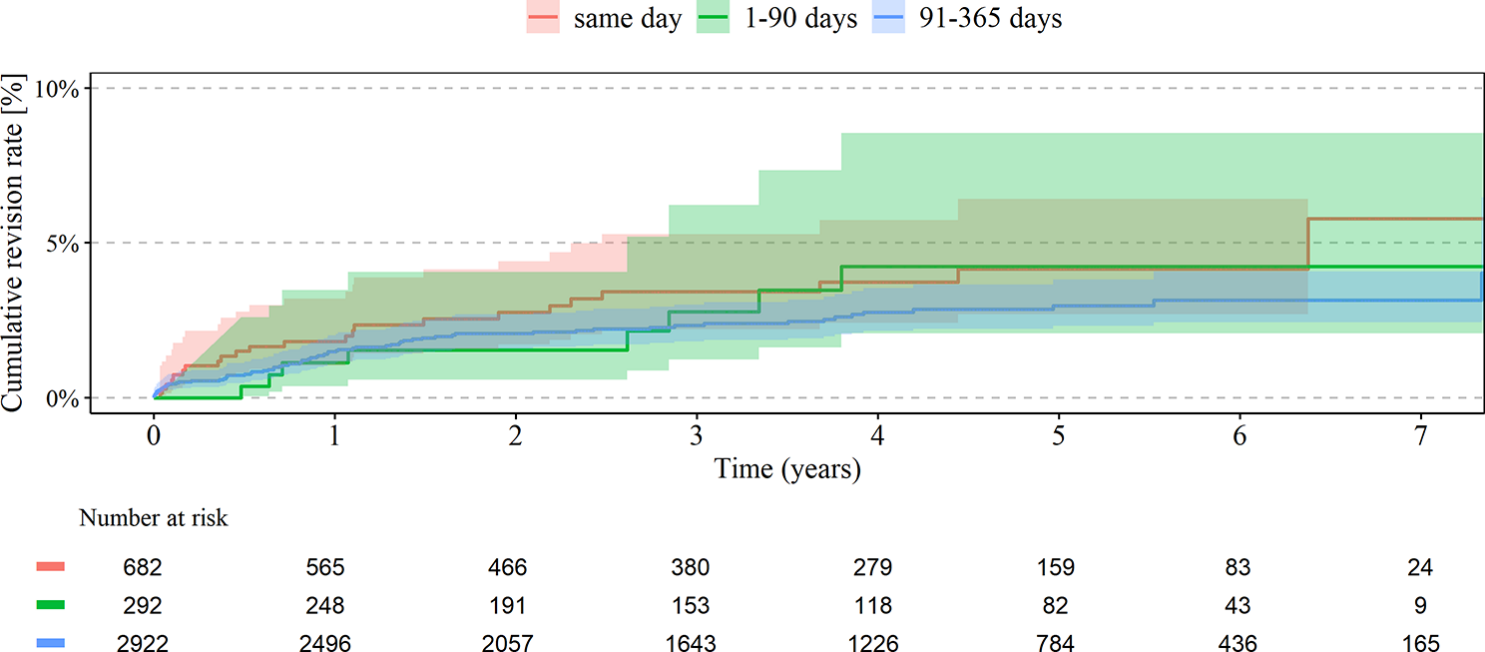
Revision rate in UKA

**Fig. 5:**
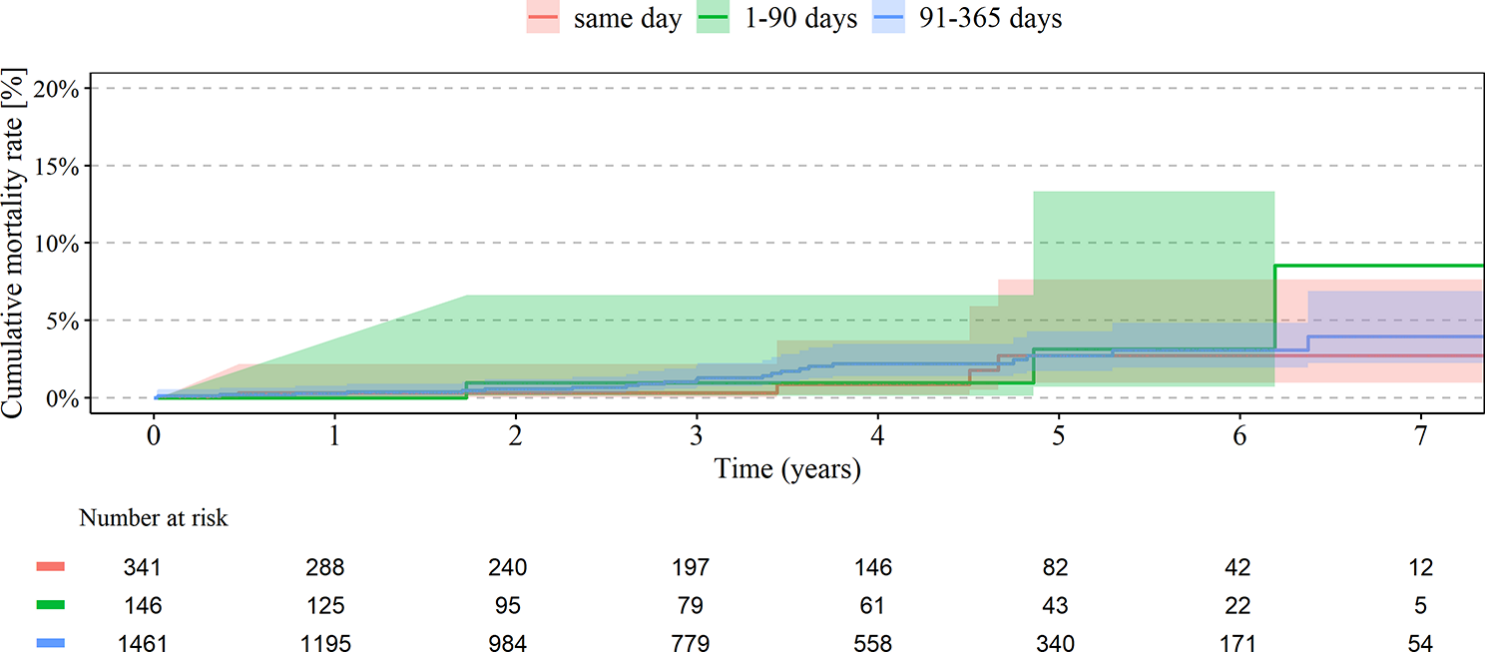
Mortality rate in UKA

Overall, UKA patients were younger and had less comorbidities. The cumulative revision rates within 7 years for UKA were not significantly different between simultaneous UKA (5.8%) compared to short (4.2%) and intermediate interval (3.1%), see Table 2; Fig. 4. There was no significant association between revision rates and cofactors. The cumulative mortality rates within 7 years for UKA were not significantly different between the three groups, s. Table 3; Fig. 5. Higher risk for mortality in bilateral UKA was seen in men (HR 2.24; 95% CI 1.02–4.93) and higher weighted Elixhauser Index (HR 1.14; 95% CI 1.04–1.26), see Table 4.

**Table 3:** Cumulative events for death of patient after second *TKA* and *UKA* (95% confidence Interval)

TKA	**1 year**	**3 years**	**5 years**	**7 years**
same day (%)	0.9 (0.4–2.3)	1.8 (0.9–3.6)	8.0 (4.9–12.8)	11.6 (7.2–18.6)
1–90 days (%)	1.5 (0.6–3.6)	2.8 (1.4–5.5)	5.5 (3.0-9.9)	13.5 (6.6–26.6)
91–365 days (%)	0.7 (0.5–0.8)	3.5 (3.1–3.9)	7.9 (7.2–8.6)	13.5 (12.1–14.9)
**UKA**	**1 year**	**3 years**	**5 years**	**7 years**
same day (%)	0.3 (0.0-2.2)	0.3 (0.0-2.2)	2.7 (1.0-7.6)	2.7 (1.0-7.6)
1–90 days (%)	0.0 (0.0.)	1.0 (0.1–6.6)	3.2 (0.7–13.3)	8.5 (2.3–29.5)
91–365 days (%)	0.3 (0.1–0.8)	1.2 (0.7–2.1)	2.7 (1.7–4.3)	4.0 (2.3–6.9)

**Table 4:** Cox regression analysis

	revision						mortality					
	TKA			UKA			TKA			UKA		
Variable	HR	95% CI	*p*	HR	95% CI	*p*	HR	95% CI	*p*	HR	95% CI	*p*
Simultaneous surgery	1.00			1.00			1.00			1.00		
1–90 d	0.42	0.20–0.90	**0.026**	0.60	0.24–1.49	0.3	1.03	0.53–1.99	> 0.9	1.69	0.37–7.72	0.5
31–365 d	0.58	0.39–0.85	**0.006**	0.63	0.38–1.05	0.075	1.22	0.80–1.87	0.3	0.85	0.28–2.58	0.8
Female sex	1.00			1.00			1.00			1.00		
Male sex	1.17	0.96–1.41	0.11	0.89	0.59–1.34	0.6	1.81	1.56–2.11	**< 0.001**	2.24	1.02–4.83	**0.045**
Age ad admission	---	---		---	---		1.08	1.07–1.09	**< 0.001**	1.05	1.01–1.10	**0.020**
Age < 65 yrs	1.00			1.00			---	---		---	---	
Age 65–74 yrs	0.61	0.49–0.77	**< 0.001**	0.78	0.48–1.27	0.3	---	---		---	---	
Age 75–84 yrs	0.75	0.59–0.96	**0.024**	0.83	0.42–1.63	0.6	---	---		---	---	
Age 85 + yrs	0.97	0.53–1.76	> 0.9	3.37	0.45–25.4	0.2	---	---		---	---	
BMI normal	1.00			1.00			1.00			1.00		
underweight	0.00	0.00-Inf.	> 0.9	0.00	0.00-Inf.	> 0.9	1.95	0.27–14.2	0.5	6.41	0.00-Inf.	> 0.9
pre-obese	0.93	0.64–1.37	0.7	0.92	0.38–2.22	0.9	0.73	0.52–1.04	0.086	29.874	0.00-Inf.	> 0.9
obese 1	0.90	0.61–1.34	0.6	1.28	0.54–3.04	0.6	1.10	0.77–1.56	0.6	21.583	0.00-Inf.	> 0.9
obese 2	1.09	0.71–1.67	0.7	1.76	0.71–4.40	0.2	1.21	0.79–1.85	0.4	31.563	0.00-Inf.	> 0.9
obese 3	1.09	0.68–1.74	0.7	1.37	0.42–4.45	0.6	2.10	1.34–3.29	**0.001**	255.621	0.00-Inf.	> 0.9
missing	0,98	0.68–1.42	> 0.9	0.85	0.36–2.04	0.7	0.89	0.65–1.22	0.5	80.260	0.00- Inf.	> 0.9
Higher Elixhauser Index	1.03	1.01–1.06	**0.006**	0.99	0.93–1.06	0.7	1.06	1.04–1.07	**< 0.001**	1.14	1.04–1.26	**0.007**
volume 1-250/y	1.00			1.00			1.00			1.00		
251–500/y	0.81	0.64–1.02	0.079	0.88	0.49–1.57	0.7	0.93	0.78–1.13	0.5	1.31	0.49–3.52	0.6
> 500/y	0.77	0.60–0.99	**0.040**	0.83	0.50–1.37	0.5	0.73	0.59–0.90	**0.004**	0.44	0.19–1.02	0.055

HR = Hazard Ratio; CI = Confidence Interval; p = p-value; BMI = Body Mass Index; normal = 18.5-24.99; underweight = < 18.5; pre-obese = 25.0-29.99; obese 1 = 30.0–34.00; obese 2 = 35.0-39.99; obese 3 = > 40

## Discussion

In this large registry cohort, revision rate was lower in two-staged bilateral TKA compared to simultaneous surgery. In several studies benefits as well as risks of simultaneous TKA compared to two-staged surgeries at different intervals have been compared. Different advantages and disadvantages are consistently reported: On the one hand there is a reduction of cumulated operating time [26] and length of stay [20] with lower costs [19, 24]. But higher complication rates like thromboembolic and neurological complications [1, 2, 19, 20, 28], and revision rates [1, 19] as well as higher blood transfusion rates [2, 9, 26] were reported. Patients undergoing same day TKA were often younger [1, 13, 27, 28] and had less comorbidities [1, 26, 28], which was not confirmed in this large German cohort.

While the registry data used for this study contained only major complications (revision and death), different studies reported about heterogenous results regarding risk for complications. Previous studies with different study designs and control groups reported conflicting results, including lower rates of deep infection and revision in simultaneous TKA [9], no difference in complication rates after six months in the data of the New Zealand National Joint Registry [13] or higher rates of complications [1–3, 19]. The conclusion in these studies was, that simultaneous TKA may be an option for healthy individuals. The mortality up to one year was low in all groups, but higher in TKA than UKA. Male sex, morbid obesity and higher Elixhauser Index were associated with higher mortality rates in TKA and UKA which should be taken into consideration by surgeons planning bilateral knee arthroplasty. A few studies demonstrated no significant difference in mortality between simultaneous and staged TKA in single-center-studies [2, 11, 22] or matched case-control studies [19]. In meta-analysis higher mortality rates after 30 or 90 days were reported [9, 20]. That might be an expression for the specialized setting of those reporting single-centers, in which all healthcare professionals will be familiar with the whole treatment process of a same day bilateral surgery. Especially for total hip arthroplasties (THA) Partridge et al. recommended a threshold of at least five same day THA per year [23] for a safe procedure. This might be applicable to TKA as well.

No difference was found in revision rates for UKA. We found slightly higher proportion of male patients in simultaneous UKA, what could be an expression of patients who are more likely to be willing to take the higher risks and challenge of the strenuous rehabilitation. Another finding is the high proportion of simultaneous UKA (18% of all bilateral UKA, compared to 4% of all bilateral TKA). This could be explained by the less invasive procedure of UKA, which makes it easier for surgeons to tend to same day surgery. Actually, there are no recommendations from the large worldwide joint registries about one- or two-staged UKA. Two meta-analyses demonstrated no significant differences in all-cause complications between simultaneous and staged bilateral UKA. The authors recommend simultaneous UKA as a suitable option for bilateral uni-compartimental knee OA due to its superior cost-effectiveness without affecting the quality of outcome [7, 17].

Just 26.1% of all observed simultaneous bilateral surgeries were performed in a high-volume center with more than 500 TKA resp. 150 UKA per year. As there was a lower risk for revision in TKA in high-volume centers, the specialization and routinely performance of these procedures seems beneficial.

## Limitations

The strength of the current study is its sample size, which is higher than all published meta-analysis and registry studies. Furthermore, the nearly complete follow-up allows for valid data on major complications. The registry setting covers real-world data and not only results from specialized centers. This study design has some limitations. Only mortality and revision rates could be analyzed, which do not give an overall picture of all peri- and post-operative complications. Data on specific complications leading to revisions are only available for such revisions which are directly entered into EPRD database. The main advantage of EPRD is the linkage between data from hospitals and from insurance companies which results in a nearly complete capture of revisions. As the data from insurance companies can only be divided by ICD/ICPM codes, we can give very reliable data on revision due to infection and due to aseptic causes. However, the retrospective data collection introduces selection bias, as group assignment was based on available data rather than randomization, potentially leading to an under- or overestimation of effects. Patients with bilateral OA, who died after their first and before the second TKA, were not included. There is an inevitable time bias like in all studies about this topic. Due to the voluntary nature of the EPRD, around 30% of the arthroplasties are missing, particularly from non-participating hospitals. Despite these limitations, the data remains broadly representative, covering approximately 70% of all arthroplasties performed in Germany.

## Conclusion

The lowest risk for revision in bilateral TKA was seen for high-volume centers, patients younger than 75 years and two-staged surgery. No differences were found in timing of UKA. Therefore, simultaneous UKA seems to be a safe option in bilateral unicondylar OA, whereas simultaneous TKA should be performed simultaneously only in selected patients in experienced centers.

## Electronic supplementary material

Below is the link to the electronic supplementary material.

Supplementary Material 1

Supplementary Material 2

## Data Availability

All data generated or analyzed during this study are included in this published article.

## Abbreviations

CI: Confidence interval
ICD-10: International classification of disease
OA: Osteoarthritis
HR: Hazard Ratio
THA: Total Hip Arthroplasty
TKA: Total Knee Arthroplasty
TJA: Total Joint Arthroplasty
