# Supplementary figures and images for: Best timing of bilateral knee arthroplasty– an analysis of revision and mortality rates from the German Arthroplasty Registry (EPRD)
Source: BMC Musculoskelet Disord. 2025 Mar 31;26:311. doi: 10.1186/s12891-025-08548-5 (PMC11956241; doi:10.1186/s12891-025-08548-5)

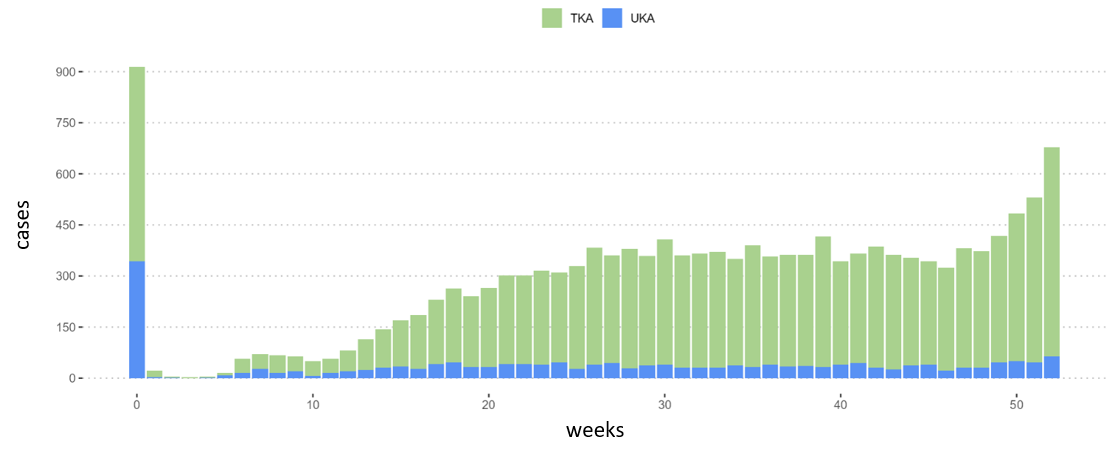

Supplement: Supplementary file 1 — Supplementary Material 1 [file 12891_2025_8548_MOESM1_ESM.png]

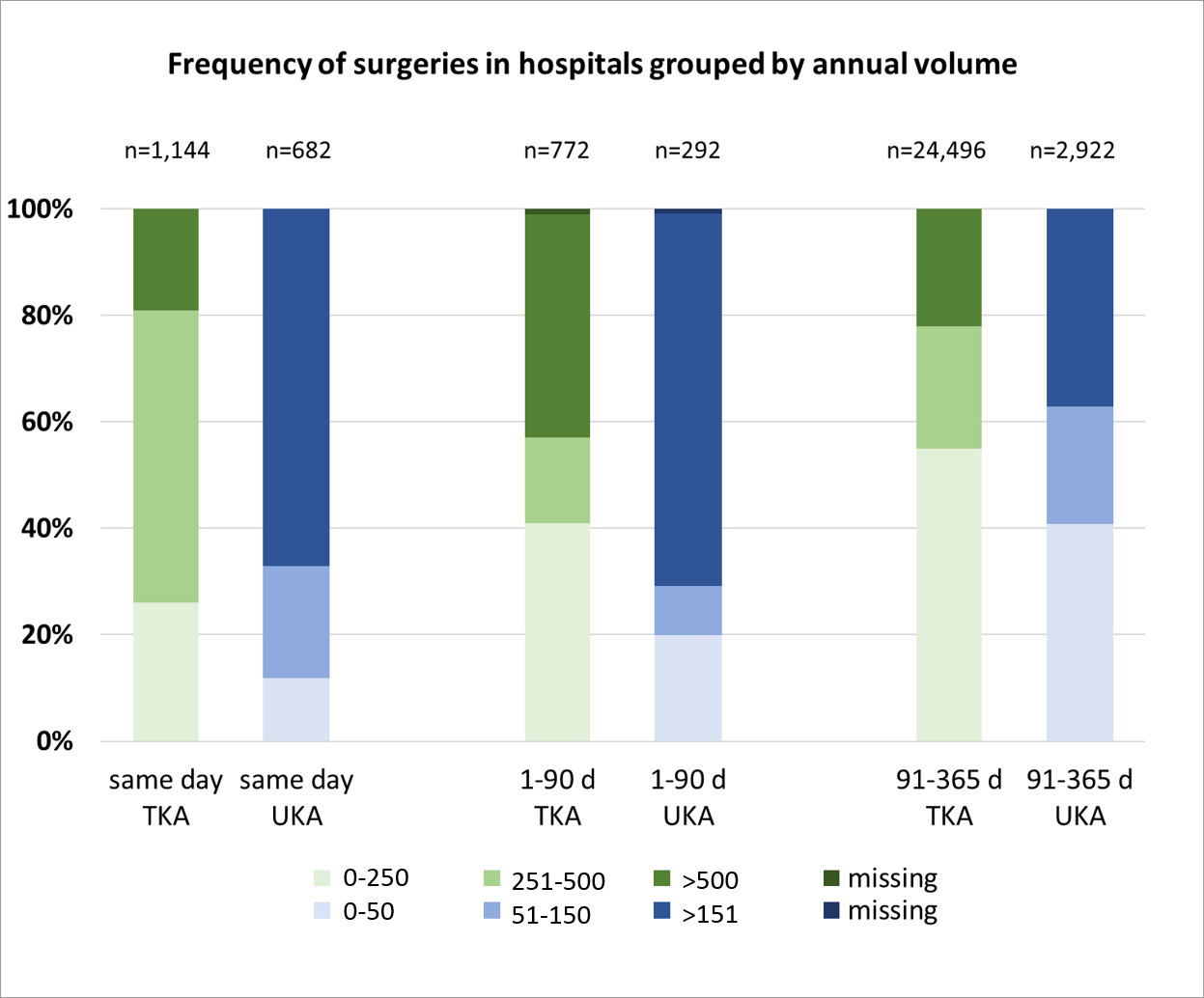

Supplement: Supplementary file 2 — Supplementary Material 2 [file 12891_2025_8548_MOESM2_ESM.png]
